# Delayed Onset of an Intradural Epidermoid Tumor in the Lumbar Region Seven Years After Spinal Anesthesia for Childbirth: A Case Report

**DOI:** 10.7759/cureus.10517

**Published:** 2020-09-17

**Authors:** Patrick Graupman, Eric S Nussbaum, Hemant Mishra

**Affiliations:** 1 Neurosurgery, Gillette Children’s Hospital, St. Paul, USA; 2 Neurosurgery, United Hospital, National Brain Aneurysm and Tumor Center, Minnesota Neurovascular and Skull Base Surgery, Minneapolis, USA; 3 Department of Veterinary and Biomedical Sciences, University of Minnesota, Minneapolis, USA

**Keywords:** epidermoid cyst, spinal puncture, spinal anesthesia

## Abstract

Epidural or spinal anesthesia is commonly administered in births in the US, and the potential risks for epidermoid tumors are not well-characterized. We present the case of a 29-year-old female patient who developed an intradural epidermoid tumor in the lumbar spine, discovered seven years after spinal anesthesia for childbirth. MRI revealed a 4 cm tumor filling the entire spinal canal. Pathology confirmed the mass to be an epidermoid. Complete surgical resection of the intradural lesion was accomplished with full symptomatic relief. This case supports the relationship between delayed development of epidermoid tumors and spinal puncture in adult populations.

## Introduction

Epidermoid tumors comprise less than 1% of spinal tumors. Previous case reports have established some epidermoid tumors as a known rare complication of lumbar puncture with most cases being described in children [1,2]. Epidural or spinal anesthesia is administered in up to 73% of births in the US [3], and the potential risks for epidermoid tumors are not well-characterized, although believed to be rare. We report a case of a 29-year-old woman presenting with an intradural lumbar epidermoid tumor, seven years after spinal anesthetic administration during childbirth. This case expands on prior evidence that lumbar spinal puncture can be complicated by delayed development of epidermoid tumors in adults.

## Case presentation

**Figure 1:**
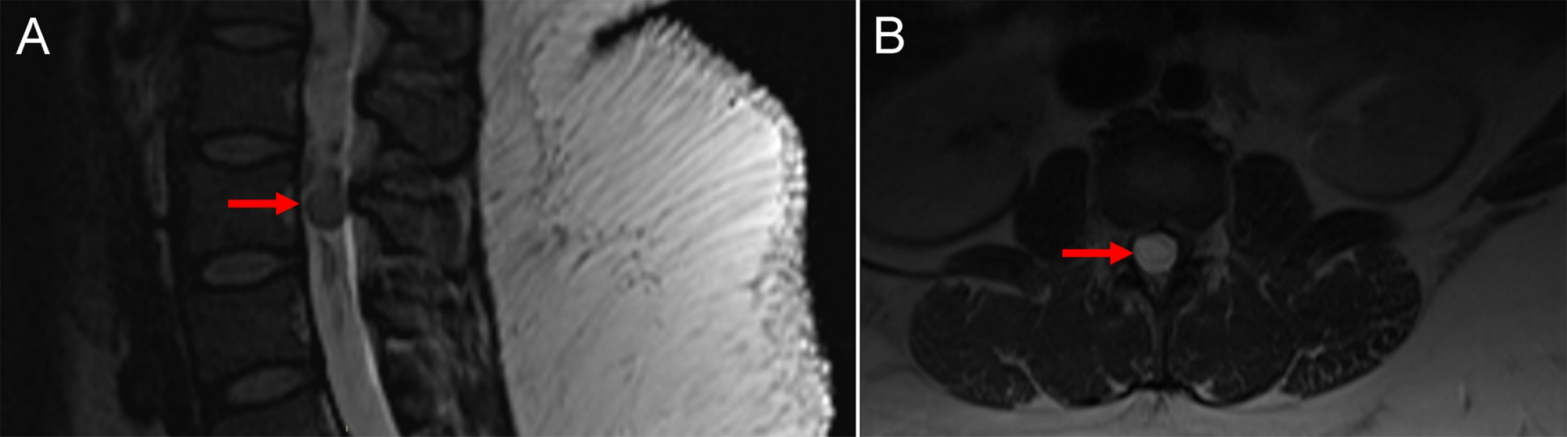
Contrasted lumbar spine MRI A) Diagnostic sagittal MRI of the lumbar spine showing a 4 cm long tumor cyst occupying the entire synovial area of the spinal canal. B) Diagnostic axial MRI at the site of the lesion. The nerve roots are not visible as they are compressed to the side of the lesion.

**Figure 2:**
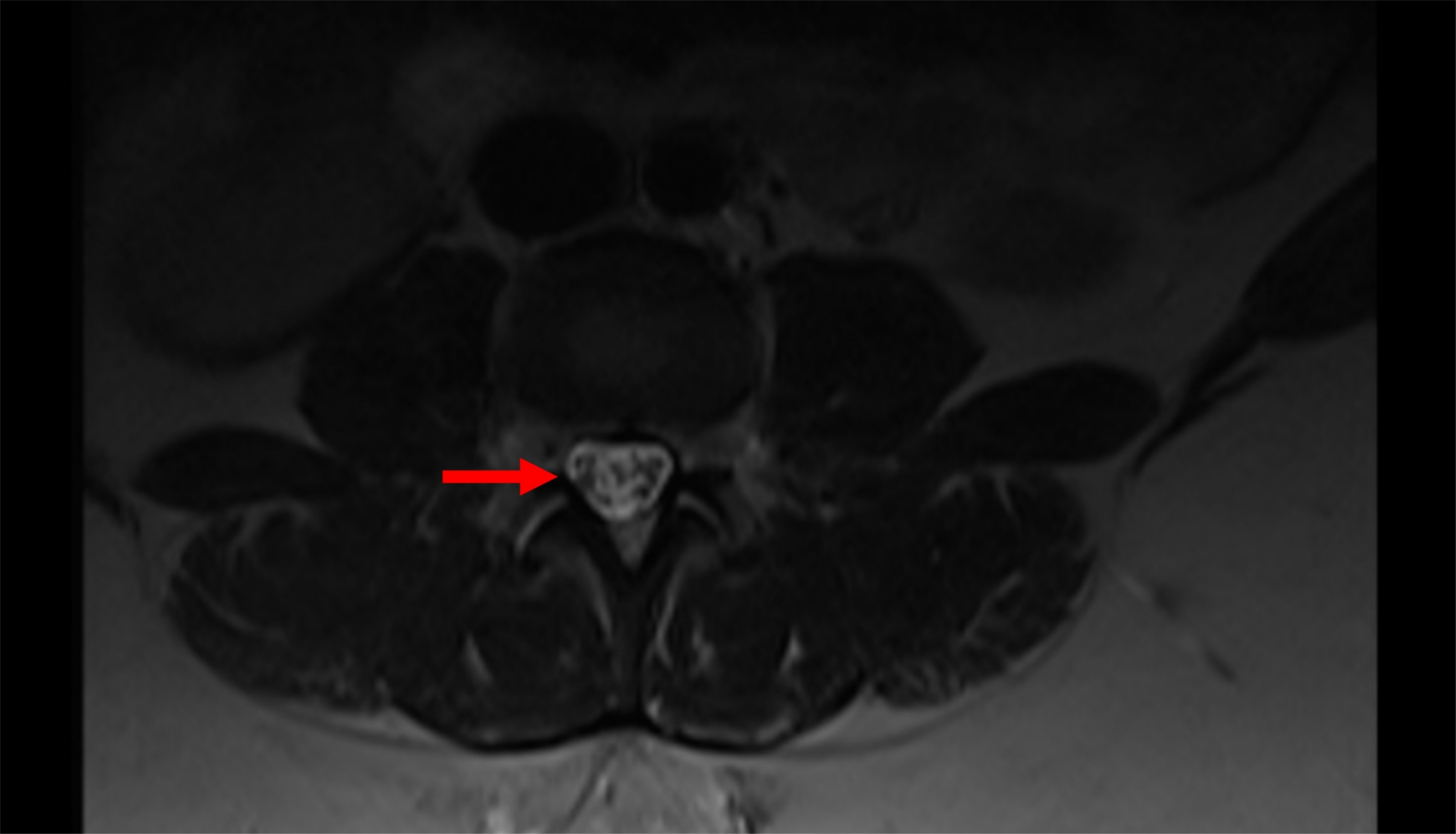
Axial MRI Image Axial normal image 2 cm below the lesion, showing the normal position of the nerve roots.

**Figure 3:**
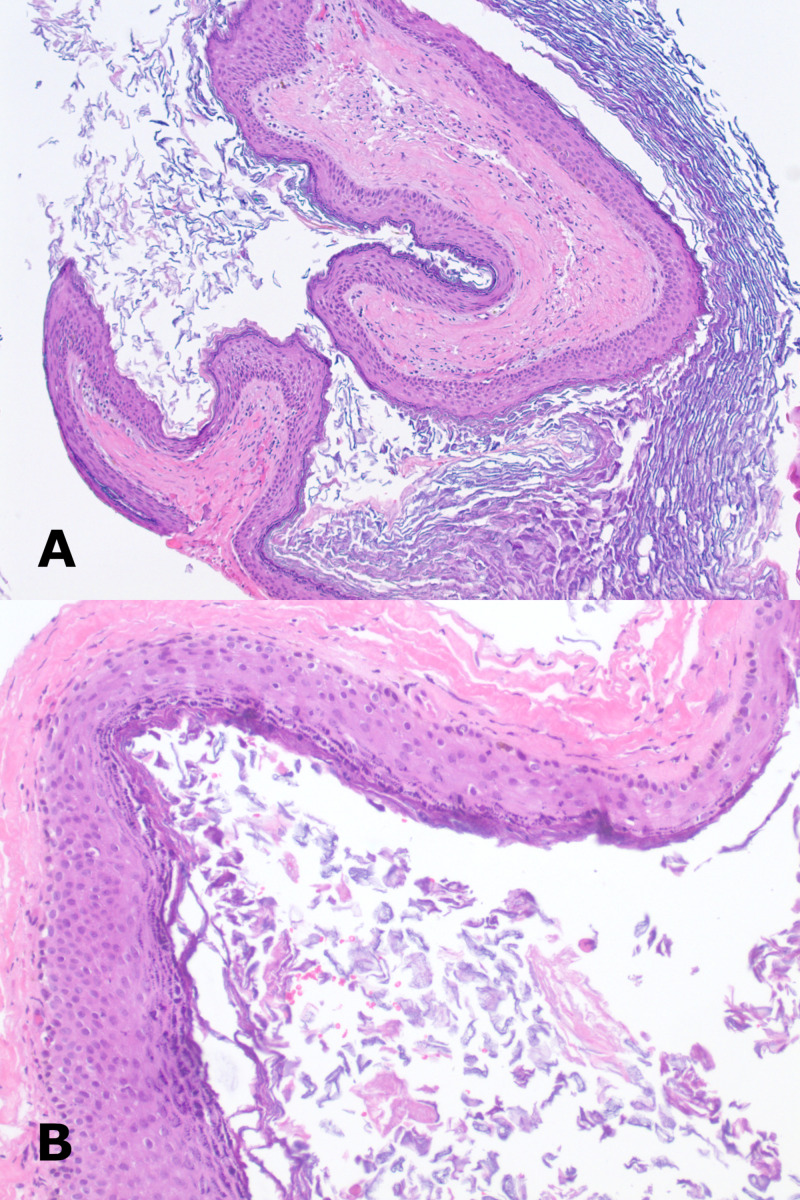
Histopathological images of the intradural epidermoid tumor A) Squamous epithelial cyst containing abundant keratin (H&E stain, original magnification: 100X). B) Squamous epithelium lacks skin adnexal structures and shows no cytologic atypia (H&E stain, original magnification: 200X).

A 29-year-old female presented with increasing back, groin, and bilateral leg pain over a six-month period. She had no bowel or bladder involvement and no weakness. The patient had no relevant past medical history but had received spinal anesthesia administered with a styleted spinal needle during childbirth seven years prior to clinical presentation. Contrasted lumbar spine MRI revealed a 4 cm long space occupying lesion without enhancement filling the spinal canal (Figures 1-2). A lumbar laminectomy was performed, and total resection of the mass achieved. Histopathological analysis revealed that the mass was a benign tumor of epidermoid origin (Figure 3). Post-operatively, the patient experienced no complications at eight-week follow-up with complete resolution of symptoms.

## Discussion

We report a rare case of an epidermoid tumor of the lumbar spine associated with lumbar puncture presenting in a delayed fashion in an adult, which was treated with complete surgical resection of the intradural lesion and full symptomatic relief was achieved. This case supports the relationship between delayed development of epidermoid tumors and spinal puncture in adult populations.

Manno et al. reviewed 90 cases of intraspinal epidermoid tumors and found that the vast majority occur in the intradural space of the lumbar spine (83/90 [92%]) [4]. A recent large review of the literature by Beechar et al. including 65 spinal epidermoid tumor cases showed that 46% are iatrogenic, and are more common in females (36/65 [55.4%]) [1]. The duration between spinal anesthesia and onset of symptoms is unpredictable, and diagnosis of cyst has been reported between three months to 10 years [5,6]. Pear et al. described an epidermoid cyst that developed 10 years after lumbar puncture for the treatment of poliomyelitis [6]. Of cases that report outcome data, the majority achieved gross total resection (44/59 [74.6%]) and good clinical outcome (54/61 [88.5%]). Epidermoid tumors arising as a consequence of spinal anesthesia during childbirth are rare. Manzo et al. reported a case of a 36-year-old female patient who, three years after receiving epidural anesthesia for Cesarean section, developed radiating back pain due to an iatrogenic lumbar epidermoid cyst [7]. The patient underwent a laminectomy, and the tumor was completely resected. Though the reported cases are heterogeneous regarding cause and location, a commonality exists in the introduction of epidermal cells into the intradural space because of lumbar puncture.

Approximately 40% of iatrogenic epidermoid spinal tumors are late complications due to lumbar puncture [2]. However, the onset of tumor symptoms caused by lumbar puncture is typically long after procedures are performed, with a mean reported latency of nine years, consistent with the delayed latency in the present case [8]. Many lumbar puncture-related epidermoid tumors are preventable when safety measures are applied, including the use of an atraumatic spinal needle [2,9,10].

In the present case, the origin of the epidermoid tumor was likely initiated by the lumbar puncture intervention, which was done for anesthesia during childbirth. The patient presented with symptoms of back, groin, and bilateral pain. The MRI findings revealed a 4 cm lesion in the spinal canal, which was successfully resected by lumbar laminectomy. The cytopathological diagnosis confirmed a mass as a benign tumor and post-operatively the patient experienced no complications at eight-week follow-up with complete resolution of back, groin, and bilateral leg pain. Therefore, the total resection of the spinal epidermoid cyst seems to have been an effective treatment option and can result in complete resolution of all the related symptoms. Furthermore, women who undergo similar interventions during childbirth or for any other surgical procedure should be monitored for the various symptoms, which could result from the development of similar types of cysts. Importantly, proactive measures should be taken to confirm the diagnosis and surgically remove these cysts.

## Conclusions

Here, we report a rare case that suggests that lumbar puncture for spinal anesthesia during childbirth carries the risk for the development of intradural epidermoid tumors that can be diagnosed several years post-procedure, highlighting the importance of careful administration of anesthesia during childbirth by adhering to best practice methods of lumbar puncture with an atraumatic needle. 
